# Effects of a nicosulfuron-based herbicide on common carp *(Cyprinus carpio)* under laboratory conditions: pathophysiological and histopathological evaluation

**DOI:** 10.2478/jvetres-2026-0027

**Published:** 2026-05-12

**Authors:** Bartosz Bojarski, Artur Osikowski, Sebastian Hofman, Leszek Szała, Patrycja Jurecka, Agnieszka Rombel-Bryzek

**Affiliations:** 1 Department of Animal Biology and Environment, Faculty of Animal Breeding and Biology, Bydgoszcz University of Science and Technology, 85-084 Bydgoszcz, Poland; 2 Department of Animal Reproduction, Anatomy and Genomics, University of Agriculture in Kraków, 30-059 Kraków, Poland; 3 Department of Comparative Anatomy, Institute of Zoology and Biomedical Research, Jagiellonian University, 30-387 Kraków, Poland; 4 Department of Mathematics, Informatics and Cybernetics, Faculty of Chemical Engineering, University of Chemistry and Technology, Prague, 166 28 Praha 6, Czech Republic; 5 lnstitute of Ichthyobiology and Aquaculture in Gołysz, Polish Academy of Sciences, 43-520 Zaborze, Poland; 6 Department of Clinical Biochemistry and Laboratory Diagnostics, Faculty of Medicine, University of Opole, 45-052 Opole, Poland

**Keywords:** fish, herbicide, blood parameters, organ microstructure, toxicity

## Abstract

**Introduction:**

Contamination with herbicides is a frequent environmental issue. The aim of the study was to evaluate haematological and blood biochemical parameters, and the microstructure of selected organs in control and herbicide-exposed fish.

**Material and Methods:**

In this study, 108 common carp (*Cyprinus carpio*) were exposed to a commercial herbicide with nicosulfuron as its active substance. Three groups of fish were established: control, TAM1 (nicosulfuron at 1 mg/L), and TAM2 (nicosulfuron at 5 mg/L). Treatment lasted 1, 3 or 10 days. Functional (pathophysiological) and structural (histopathological) alterations in control and herbicide-exposed fish were evaluated.

**Results:**

Various haematological changes and some biochemical alterations were detected. The pathophysiological changes suggested erythrocyte damage, compensatory response (as indicated by an increase in red blood cell count and erythroblast percentage), stress response (as evidenced by an increase in glucose concentration) and possible lipid metabolism disorders (as indicated by a decrease in cholesterol concentration). More differences in pathophysiological parameters were detected between TAM2 fish and control fish than between TAM1 fish and control fish. Histopathological analysis revealed renal tubule necrosis, of which the severity depended on herbicide concentration and exposure duration.

**Conclusion:**

The exposure of common carp to the tested nicosulfuron-based herbicide formulation led to pathophysiological and histopathological alterations, indicating the need for systematic detection and quantification of nicosulfuron in aquatic environments.

## Introduction

Herbicides are agents used for weed control and constitute the most prevalent category of pesticides, representing approximately 50% of all crop protection chemicals used worldwide (16, 18). Their extensive use leads to contamination of the aquatic environment. Herbicide contamination of water ecosystems has emerged as a significant issue in many countries and has been documented in Australia (3), Brazil (17), Canada (25, 46), China (26), Croatia (24), Italy (35), Poland (21) and Sweden (33).

Nicosulfuron is a post-emergence sulfonylurea herbicide used to control annual broadleaved weeds and grasses in maize crops. It is a selective systemic herbicide absorbed through foliage and roots and translocated throughout the plant. It inhibits amino acid synthesis by suppressing acetohydroxyacid synthase activity (31). The nicosulfuron contained in some herbicide formulations may enter the aquatic environment, and the compound has been detected in water samples from the Warta River in Poland (29) as well as in influent wastewater in Greece (42). Its half-life in agricultural pond water is 75 days, indicating relatively high stability in the aquatic environment (14).

Exposure of fish to herbicides may result in stress response, liver and kidney dysfunction and immunosuppression (11). According to Bojarski *et al*. (6), herbicides cause changes in various physiological parameters in fish, such as reduced acetylcholinesterase activity in internal organs, increased hepatic transaminase activities and disturbances in redox balance. Alterations in haematological parameters usually indicate anaemia and inflammatory processes. The most frequently observed histopathological changes include hyperplasia and hypertrophy of the gill epithelium, as well as changes in liver microstructure. To date, few scientific studies on the toxicity of nicosulfuron to fish have been published. Bretaud *et al*. (13) showed that goldfish (*Carassius auratus*) treated with nicosulfuron exhibited decreased brain acetylcholinesterase (AChE) activity. Saglio *et al*. (44) demonstrated that exposure to nicosulfuron caused behavioural changes in fish of the same species. Adene *et al*. (1) reported that exposure to this herbicide led to behavioural and haematological alterations in African catfish (*Clarias gariepinus*). In contrast, research conducted by Li *et al*. (32) showed that the toxicity of nicosulfuron to zebrafish (*Danio rerio*) embryos was low.

The common carp (*Cyprinus carpio* Linnaeus, 1758) is a very important aquaculture species in many Asian and some European countries (40). Yancheva *et al*. (53) stated that this species is as important for aquatic toxicological research as it is for aquaculture. They noted that its usefulness in ecotoxicology has been confirmed through various laboratory experiments, field studies and biomonitoring programmes. The common carp was marked as a valuable species for haematological and toxicological research also in the experience of the present researchers. It was decided to use this species because it is a suitable model for such research and also because its biology is well studied and characterised.

Haematological assessment is a widely accepted method for monitoring fish health and detecting physiological alterations induced by exposure to organic and inorganic substances. Therefore, it is applicable in both ecotoxicology and pharmacotoxicology (52). Haematological analysis is often complemented by biochemical testing of blood plasma or serum. Blood-based biochemical analyses are widely used as predictive and diagnostic tools for assessing fish welfare in both aquaculture and research, including studies in fish toxicology (12). Alterations in the microstructure of organs can serve as biomarkers of effects induced by toxicants. Histopathological alterations in organs such as gills, liver and kidneys have been successfully used in ecotoxicological studies and risk assessment programmes (54). Bojarski *et al*. (8) highlighted that the simultaneous use of haematological analysis, blood biochemical testing and histopathological examination enables comprehensive evaluation of the effects of toxic agents on fish. In the present study, the potential toxic effects of the nicosulfuron-based herbicide formulation Tamizan 040 OD on common carp were assessed. In order to detect both functional (pathophysiological) and structural (histopathological) alterations, haematological and blood biochemical parameters and the microstructure of the gills, liver and kidney were evaluated in control and herbicide-exposed fish.

## Material and Methods

### Experiment design

In the present study, 108 healthy sexually immature individuals of both sexes of common carp of the R3R8 laboratory line were used. The body weight of the fish was 68.76 ± 5.74 g (mean ± SD), and the total length was 16.12 ± 0.57 cm (mean ± SD). Fish were placed randomly in nine indoor tanks, three allocated to each group (12 fish per tank). Each tank had a volume of 300 L and was filled with water to a volume of 200 L. The experiment commenced with a 14-day acclimation period. After this period, the fish were divided into three equal groups: control (C) and two experimental groups (TAM1 and TAM2). The fish in group C were kept in water without the addition of the tested herbicide formulation. The fish in group TAM1 were exposed to the commercial herbicide Tamizan 040 OD (Synthos Agro, Oświęcim, Poland) at a low concentration, corresponding to 1 mg/L of the active ingredient (nicosulfuron), while the fish in group TAM2 were exposed to the same herbicide formulation at a higher tested concentration, corresponding to 5 mg/L of the same active compound. As no analytical verification of the actual concentrations was performed in the present study, the values given above should be considered nominal. The exposure lasted 1, 3 or 10 d. After these periods, biological material was collected and the fish were not subjected to further treatment.

### Experimental conditions

During acclimation, the water in each tank was replaced every 12 h to habituate the fish to regular water changes and remove nitrogenous metabolites. During the exposure period, the water in the control group and the herbicide solution in the experimental groups were also replaced every 12 h to eliminate nitrogenous metabolites and maintain a constant herbicide concentration. Water quality parameters (*i.e*. the concentrations of dissolved oxygen, ammonia, nitrites and nitrates and the pH, general hardness, carbonate hardness and temperature) were measured every 48 h during both acclimation and exposure. The temperature and dissolved oxygen levels were measured using an Oxi 3310 oximeter (WTW, Weilheim, Germany), and pH was assessed with a pH meter (Mettler Toledo, Greifensee, Switzerland). Ammonia, nitrite and nitrate concentrations, general hardness and carbonate hardness were determined using colorimetric test kits manufactured by Zoolek (Łódź, Poland). A photoperiod of 14 h light and 10 h darkness was maintained throughout the current experiment. The fish were fed with Aller Silver 3 mm (Aller Aqua, Christiansfeld, Denmark) once a day. No feed was provided on the days when euthanasia was performed.

### Biological material collection

After each exposure period (1, 3 and 10 d), biological material was collected from 12 individuals from each group. Blood was sampled from 12 fish, and the organs were collected from 6 randomly selected individuals. Blood was sampled from the caudal vein with a needle and syringe into heparinised tubes (Heparinum WZF; WZF Polfa, Warsaw, Poland) without the use of anaesthetic, as it is known that anaesthetics can affect haematological (5, 43, 51) and blood biochemical parameters (23, 30, 43, 48, 49). Some of the collected blood was subjected to haematological analysis immediately after sampling, and the rest was centrifuged for 20 min (3,000 × *g*) using a Mikro 200R centrifuge (Hettich, Tuttlingen, Germany). The obtained plasma was stored frozen (−80°C) until biochemical analyses were performed. After the blood sampling, the fish were euthanised by administration of MS 222 (500 mg/L; Merck, USA). Next, the gills, liver and trunk kidney were collected.

### Haematological analysis

In order to determine the RBC and WBC counts, the blood was diluted 100-fold with Hayem’s solution. The cells were then counted using a Bürker chamber and a standard optical microscope. For HCT determination, capillary tubes were filled with blood (to about ¾ of their volume) and then centrifuged for 5 min (16,000 × *g*). Then, the percentage of the erythrocyte layer was evaluated with a standard reader. For determination of HGB concentration, the blood was mixed with Drabkin’s reagent (1 : 250 ratio; Chempur, Piekary Śląskie, Poland). The absorbance was read at the 540 nm wavelength using a spectrophotometer. Calculations of MCV, MCH and MCHC were made using standard formulae. Blood smears were also prepared and stained with a Hem-Kolor kit (Stamar, Dąbrowa Górnicza, Poland). Next, a leukogram (differential leukocyte count) and an erythrogram (characterisation of erythrocyte morphology on the basis of analysis of a stained blood smear) were determined at a magnification of 1,000×. In each smear, 100 leukocytes for the leukogram and 300 erythrocytes for the erythrogram were inspected. Analysis of the erythrogram identified normal mature erythrocytes, normal erythroblasts, abnormal erythrocytes with altered cell shape, abnormal erythrocytes with altered nucleus shape and haemolysed erythrocytes. Each haematological determination was conducted for n = 12. Detailed methodological information regarding the haematological analysis was provided in our previous article (9).

### Biochemical analysis

Blood plasma was analysed in order to assess glucose, total protein, triglyceride and total cholesterol concentrations, as well as ALT activity. The biochemical indicators were determined using BioSystems kits (Barcelona, Spain) according to the manufacturer’s instructions; the only modification to the procedure involved calculating ALT activity based on two absorbance measurements instead of four, as described by Bojarski *et al*. (8). Determination of the non-enzymatic biochemical parameters (glucose, total protein, triglyceride and total cholesterol concentrations) was conducted with an Epoch microplate reader (BioTek Instruments, Winooski, VT, USA), and ALT activity was measured with a V-730 UV-visible spectrophotometer (Jasco, Tokyo, Japan). The biochemical determinations were conducted for n=12, except for the determination of triglyceride concentration in group TAM2 after 3 d of exposure, where n was equal to 10 due to technical issues.

### Histopathological analysis

Tissues for the histological analysis were taken from six individuals of each of the examined groups at each sampling time. Samples of gills, livers and trunk kidneys were prepared using the standard formalin-fixed, paraffin-embedded technique and routinely stained with HE (Delafield’s haematoxylin and eosin Y) as follows. Tissue samples were fixed in 4% buffered formaldehyde (Chempur) for two weeks and embedded in Paraplast Regular (Sigma, St. Louis, MO, USA). Transverse sections of 6 μm were prepared on a Zeiss Hyrax M55 microtome (Oberkochen, Germany), affixed to glass slides, stained with Delafield’s haematoxylin for 5 min and, after being washed in tap water, stained with eosin solution also for 5 min. The slides were blindly analysed using a light microscope (Eclipse E600). Digital images were captured using a DS-Fi1c camera and analysed with NIS-Elements F Software (all from Nikon, Tokyo, Japan). The analysis focused on the overall histological structure of the organs and identification of potential abnormalities. The methodology of histological procedures was similar to that in previous studies on similar topics (8, 10).

### Statistical analysis

The compliance of the data with the normal distribution for each parameter and each group was verified using the Shapiro–Wilk test of normality. The homogeneity of variances of the data in all the groups for each parameter was verified using the Levene homogeneity of variance test. For haematological parameters and plasma biochemical indicators, the one-way ANOVA model was applied and, if significant, was followed by the Tukey honestly significant difference test because the Shapiro–Wilk test of normality and the Levene homogeneity of variance test in most (or in all) cases did not reject their null hypotheses. For the parameters constituting the erythrogram and the leukogram, the Kruskal–Wallis test was applied and, if significant, was followed by the Dunn test with Bonferroni correction because of the results of the Shapiro–Wilk test of normality. The significance level was set at 0.05 for all the tests used. In the results of the *post-hoc* analysis (*i.e*. the Tukey HSD test and the Dunn test with Bonferroni correction), the notation P-value < 0.05 is a simplification of 0.01 ≤ P-value < 0.05; analogously P-value < 0.01 is used for 0.001 ≤ P-value < 0.010, and P-value < 0.001 signifies 0.000 ≤ P-value < 0.001. All *post-hoc* comparisons were conducted using two-sided (two-tailed) alternative hypotheses. The statistical analysis was performed in R software v. 4.5.0 (39).

## Results

### Water physicochemical analyses

The results of the water physicochemical analyses are presented in Table 1.

**Table 1.** Water parameters (mean ± SD) in the control and experimental groups

Parameter	Control group	TAM1	TAM2
Temp. (°C)	19.75 ± 0.21	19.73 ± 0.22	19.70 ± 0.21
O_2_ (mg/L)	5.52 ± 0.32	5.33 ± 0.42	5.24 ± 0.61
pH	7.31 ± 0.05	7.29 ± 0.05	7.31 ± 0.06
NH_3_ (mg/L)	0.02 ± 0.01	0.02 ± 0.01	0.01 ± 0.01
NO_2_− (mg/L)	0.11 ± 0.15	0.09 ± 0.13	0.07 ± 0.08
NO_3_− (mg/L)	12.41 ± 5.45	10.69 ± 5.30	9.83 ± 5.65
GH (dGH)	5.10 ± 0.31	5.14 ± 0.35	5.21 ± 0.41
KH (dKH)	3.00 ± 0.00	3.00 ± 0.00	3.00 ± 0.00

1 TAM1 – group intoxicated with Tamizan 040 OD nicosulfuron herbicide at 1 mg/L of the active substance; TAM2 – group intoxicated with the herbicide at 5 mg/L of the active substance; GH – general hardness; KH – carbonate hardness

### Haematological parameters

**Fig. 1.**
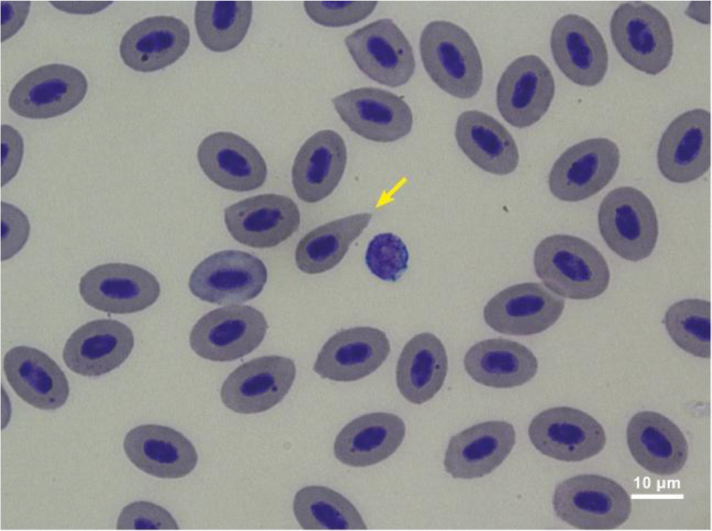
Abnormal RBC of common carp intoxicated with Tamizan 040 OD nicosulfuron herbicide for 1 day. Erythrocytes showing changed cell shape

The only statistically significant difference in the haematological parameters between the control and the experimental groups after one day of exposure was a higher percentage of erythrocytes showing altered cell shape (P-value < 0.05) (Fig. 1) in the fish exposed to the herbicide at the higher tested concentration (Table 2). The percentage of abnormally shaped red blood cells detected in fish from group TAM2 was also significantly higher than the percentage of this type of erythrocyte noted in fish from group TAM1 (P-value < 0.05). The RBC count in fish from the group treated with Tamizan at the higher tested concentration was statistically significantly higher (P-value < 0.01) than that in fish from group TAM1. Similarly, the HCT level and HGB concentration in fish from group TAM2 were significantly higher than the values noted in fish from group TAM1 (P-value < 0.001 and P-value < 0.05, respectively.

**Table 2.** Haematological changes in common carp after 1 day of exposure to Tamizan 040 OD nicosulfuron herbicide

Parameter	Control group	TAM1	TAM2
RBC (10^6^/μL)	1.53 ± 0.24	1.42 ± 0.17	1.71 ± 0.24
HCT (%)	27.54 ± 2.22	25.90 ± 1.31	29.33 ± 2.49
HGB (g/dL)	6.90 ± 0.64	6.64 ± 0.62	7.36 ± 0.79
MCV (fL)	184.14 ± 32.93	186.04 ± 28.98	174.85 ± 26.50
MCH (pg)	45.93 ± 7.34	47.76 ± 8.94	43.90 ± 7.12
MCHC (g/dL)	25.07 ± 1.82	25.61 ± 1.72	25.11 ± 1.74
MNE (%)	98.06 ± 1.19	97.69 ± 1.33	95.44 ± 3.19
EAS (%)	0.17 ± 0.27	0.14 ± 0.22	0.78 ± 0.69*
EAN (%)	0.14 ± 0.22	0.36 ± 0.44	0.19 ± 0.17
HE (%)	0.33 ± 0.40	0.67 ± 0.70	1.58 ± 1.88
EB (%)	1.31 ± 1.13	1.14 ± 0.92	2.00 ± 2.09
WBC (10^3^/μL)	30.63 ± 7.52	26.04 ± 4.87	27.69 ± 9.52
LYM (%)	95.33 ± 4.38	93.92 ± 3.58	96.33 ± 2.77
Seg (%)	1.67 ± 2.74	2.92 ± 2.11	1.83 ± 1.80
ImNeu (%)	2.58 ± 2.35	2.83 ± 1.95	1.75 ± 1.29
Mono (%)	0.42 ± 0.51	0.33 ± 0.49	0.08 ± 0.29

1 TAM1 – group intoxicated with Tamizan 040 OD nicosulfuron herbicide at 1 mg/L of the active substance; TAM2 – group intoxicated with the herbicide at 5 mg/L of the active substance;

1 MNE – mature normal erythrocytes; EAS – erythrocytes of altered cell shape; EAN – erythrocytes of altered nucleus shape; HE – haemolysed erythrocytes; EB – erythroblasts; Seg – segmented neutrophils; ImNeu – immature neutrophils; Mono – monocytes. Data are presented as means ± SD;

* – significant difference from control group value (0.01 ≤ adjusted P-value < 0.05)

The only statistically significant difference in the haematological parameters between the control and the experimental groups after 3 d of treatment was in MCH, which was lower in the fish from group TAM2 (P-value < 0.05) (Table 3). No significant differences in haematological indicators between groups TAM1 and TAM2 after 3 d of treatment were detected (P-value ≥ 0.05).

**Table 3.** Haematological changes in common carp after 3 days of exposure to Tamizan 040 OD nicosulfuron herbicide

Parameter	Control group	TAM1	TAM2
RBC (10^6^/μL)	1.08 ± 0.10	1.17 ± 0.18	1.21 ± 0.20
HCT (%)	26.00 ± 1.78	27.46 ± 1.84	25.96 ± 2.38
HGB (g/dL)	7.03 ± 0.66	7.18 ± 0.41	6.69 ± 0.76
MCV (fL)	243.02 ± 27.93	239.04 ± 39.24	218.95 ± 30.80
MCH (pg)	65.56 ± 7.51	62.38 ± 9.01	56.40 ± 8.61*
MCHC (g/dL)	27.02 ± 1.77	26.20 ± 1.38	25.78 ± 1.78
MNE (%)	97.67 ± 1.65	97.58 ± 1.46	97.08 ± 1.88
EAS (%)	0.78 ± 0.69	0.92 ± 0.81	0.81 ± 0.63
EAN (%)	0.17 ± 0.22	0.75 ± 0.75	0.94 ± 1.42
HE (%)	0.69 ± 1.04	0.11 ± 0.16	0.08 ± 0.21
EB (%)	0.69 ± 0.85	0.64 ± 0.48	1.08 ± 1.20
WBC (10^3^/μL)	25.23 ± 4.81	26.04 ± 5.17	27.04 ± 3.80
LYM (%)	94.50 ± 3.90	93.67 ± 4.48	96.50 ± 1.68
Seg (%)	2.67 ± 1.78	2.25 ± 2.09	1.25 ± 1.14
ImNeu (%)	2.75 ± 2.73	4.08 ± 3.15	2.25 ± 1.29
Mono (%)	0.08 ± 0.29	0.00 ± 0.00	0.00 ± 0.00

1 TAM1 – group intoxicated with Tamizan 040 OD nicosulfuron herbicide at 1 mg/L of the active substance; TAM2 – group intoxicated with the herbicide at 5 mg/L of the active substance;

1 MNE – mature normal erythrocytes; EAS – erythrocytes of altered cell shape; EAN – erythrocytes of altered nucleus shape; HE – haemolysed erythrocytes; EB – erythroblasts; Seg – segmented neutrophils; ImNeu – immature neutrophils; Mono – monocytes. Data are presented as means ± SD;

* – significant difference from control group value (0.01 ≤ adjusted P-value < 0.05)

**Fig. 2.**
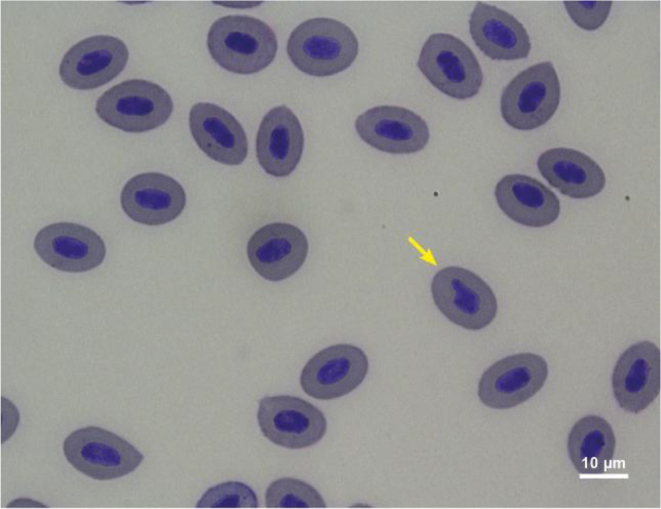
Abnormal RBC of common carp intoxicated with Tamizan 040 OD nicosulfuron herbicide for 10 days. Erythrocytes showing changed nucleus shape

After 10 d of exposure, the fish treated with the herbicide at the higher tested concentration showed significantly raised RBC count (P-value < 0.05) and significantly lowered MCH (P-value < 0.01) in comparison to the control individuals (Table 4). The MCH detected in group TAM2 was also lower than the value in group TAM1 (P-value < 0.05). The percentage of mature normal erythrocytes in the fish from group TAM2 was significantly lower (P-value < 0.01) than the percentage noted in the control individuals. Similarly, the percentage of red blood cells exhibiting altered nucleus shape (Fig. 2) was lower in group TAM2 than in the control (P-value < 0.05). However, the percentage of erythroblasts in the fish from group TAM2 was significantly higher than the percentage in the control fish (P-value < 0.001) (Table 4), and higher than the percentage in group TAM1 (P-value < 0.05).

**Table 4.** Haematological changes in common carp after 10 days of exposure to Tamizan 040 OD nicosulfuron herbicide

Parameter	Control group	TAM1	TAM2
RBC (10^6^/μL)	1.23 ± 0.23	1.29 ± 0.24	1.48 ± 0.24*
HCT (%)	26.17 ± 2.25	27.38 ± 2.56	26.77 ± 2.37
HGB (g/dL)	6.93 ± 0.48	6.97 ± 0.71	6.74 ± 0.85
MCV (fL)	220.74 ± 50.79	217.45 ± 39.96	183.34 ± 19.72
MCH (pg)	58.13 ± 11.05	55.44 ± 10.83	45.99 ± 4.56**
MCHC (g/dL)	26.54 ± 1.37	25.51 ± 2.18	25.14 ± 1.54
MNE (%)	96.89 ± 1.44	96.17 ± 1.36	94.03 ± 2.28**
EAS (%)	1.00 ± 0.86	1.36 ± 0.98	2.19 ± 1.54
EAN (%)	0.92 ± 0.89	0.47 ± 0.36	0.25 ± 0.25*
HE (%)	0.22 ± 0.30	0.53 ± 0.61	0.72 ± 1.24
EB (%)	0.97 ± 0.73	1.47 ± 0.95	2.81 ± 0.97***
WBC (10^3^/μL)	26.60 ± 6.92	22.29 ± 4.68	29.40 ± 9.03
LYM (%)	93.83 ± 3.61	93.92 ± 2.31	95.25 ± 2.56
Seg (%)	3.58 ± 2.71	2.83 ± 0.94	2.58 ± 1.88
ImNeu (%)	2.50 ± 1.45	3.25 ± 2.14	2.08 ± 1.56
Mono (%)	0.08 ± 0.29	0.00 ± 0.00	0.08 ± 0.29

1 TAM1 – group intoxicated with Tamizan 040 OD nicosulfuron herbicide at 1 mg/L of the active substance; TAM2 – group intoxicated with the herbicide at 5 mg/L of the active substance;

1 MNE – mature normal erythrocytes; EAS – erythrocytes of altered cell shape; EAN – erythrocytes of altered nucleus shape; HE – haemolysed erythrocytes; EB – erythroblasts; Seg – segmented neutrophils; ImNeu – immature neutrophils; Mono – monocytes. Data are presented as means ± SD;

*/**/*** – significant difference from control group value (0.01 ≤ adjusted P-value < 0.05 / 0.001 ≤ adjusted P-value < 0.010 / 0.000 ≤ adjusted P-value < 0.001)

No significant differences in haematological parameters between group TAM1 and the control group were detected (P-value ≥ 0.05), regardless of exposure time (Tables 2–4).

### Biochemical parameters

**Fig. 3.**
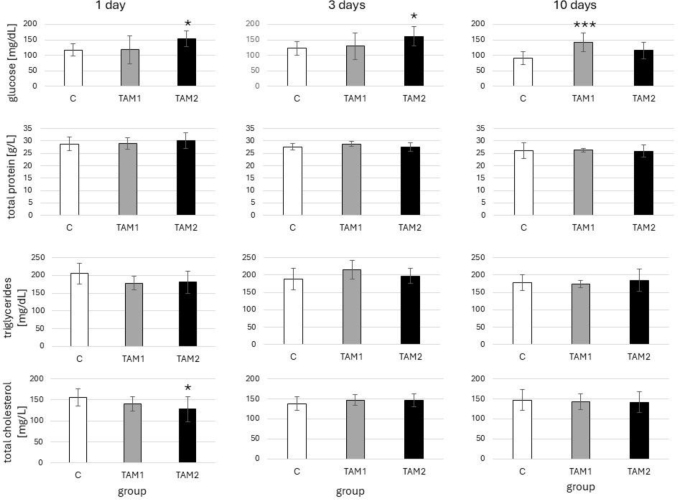
Changes in non-enzymatic plasma biochemical parameters of common carp after 1, 3 and 10 days of exposure to Tamizan 040 OD nicosulfuron herbicide. TAM1 – group intoxicated with Tamizan 040 OD nicosulfuron herbicide at 1 mg/L of the active substance; TAM2 – group intoxicated with the herbicide at 5 mg/L of the active substance. Data are presented as means ± SD; significant differences compared to the control values are marked with asterisks; */*** – significant difference from control group value (0.01 ≤ adjusted P-value < 0.05 / 0.000 ≤ adjusted P-value < 0.001)

After 1 d of exposure, the fish from the group exposed to Tamizan at the higher tested concentration had statistically significantly higher plasma glucose concentration (P-value < 0.05) than the fish from the control group (Fig. 3). Moreover, the glucose level detected in the blood plasma of fish from group TAM2 was significantly higher (P-value < 0.05) in comparison to the level noted in the case of the fish from group TAM1. Short term exposure (1 d) also led to a statistically significant decrease (P-value < 0.05) in total cholesterol concentration in the blood plasma of fish from group TAM2 in comparison to this parameter of control individuals (Fig. 3). No statistically significant differences were recorded between group TAM1 and the control group after 1-d exposure.

After 3 d of treatment, a statistically significant elevation (P-value < 0.05) in the plasma glucose concentration in the fish from group TAM2 was measured compared to the concentration in the fish from the control group (Fig. 3). No statistically significant differences in the biochemical parameters tested in the current study were recorded between group TAM1 and the control group, nor between groups TAM1 and TAM2 after 3 d of treatment.

Longer exposure (10 d) led to a statistically significantly higher (P-value < 0.001) plasma glucose concentration in the fish from group TAM1 in comparison to the control individuals (Fig. 3). No statistically significant differences in the tested biochemical parameters were recorded between group TAM2 and the control group, nor between groups TAM1 and TAM2 after 10 d of treatment.

In the current experiment, no statistically significant changes in the concentrations of total protein and triglycerides, nor in ALT activity, were observed between the individual groups, regardless of exposure duration (Fig. 3, Table 5).

**Table 5.** ALT activity in blood plasma of common carp after 1, 3 and 10 days of exposure to Tamizan 040 OD nicosulfuron herbicide

Exposure duration	Control group	TAM1	TAM2
1 day	16.43 ± 4.43	18.31 ± 15.63	17.55 ± 6.37
3 days	10.95 ± 6.56	16.44 ± 11.47	20.68 ± 10.03
10 days	12.07 ± 6.57	18.40 ± 12.99	11.24 ± 9.46

1 TAM1 – group intoxicated with Tamizan 040 OD nicosulfuron herbicide at 1 mg/L of the active substance; TAM2 – group intoxicated with the herbicide at 5 mg/L of the active substance. Data are presented as mean ± SD; no statistically significant differences between individual groups

### Histopathological analysis

**Fig. 4.**
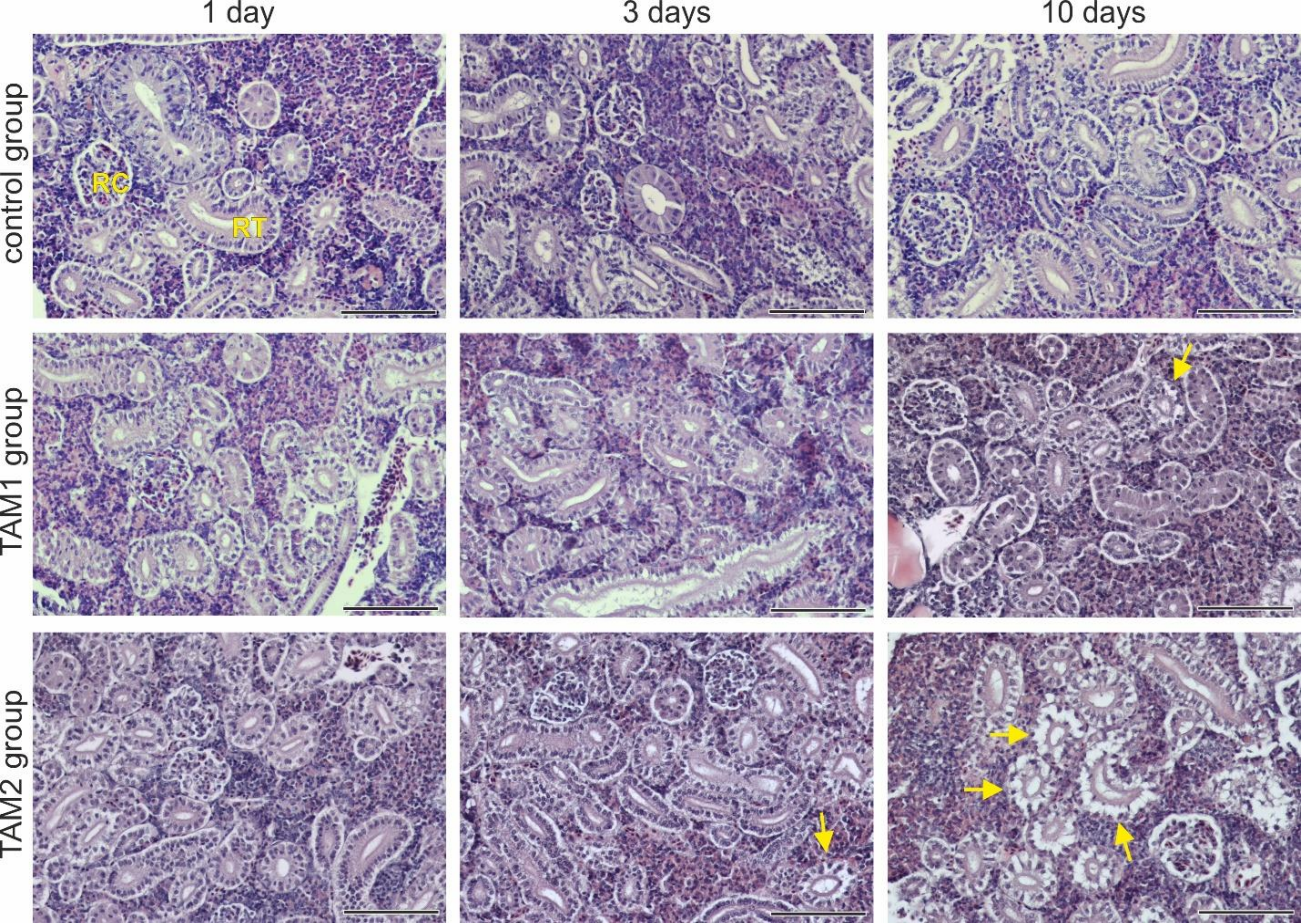
Photomicrographs of the trunk kidney of common carp in the control and experimental groups. TAM1 – group intoxicated with Tamizan 040 OD nicosulfuron herbicide at 1 mg/L of the active substance; TAM2 – group intoxicated with the herbicide at 5 mg/L of the active substance; RC – renal corpuscle; RT – renal tubule. Arrows indicate degeneration of renal tubules. Haematoxylin and eosin staining. Scale bars = 100 μm

The microstructure of the gills and liver was typical, with no histopathological changes in any of the groups. The trunk kidney (Fig. 4) also displayed a typical structure, comprising nephrons with a renal corpuscle (containing glomerulus), renal tubule, collecting duct and interstitial tissue. No histopathological changes in the trunk kidney were observed in the control specimens or in the individuals from group TAM1 after 1 and 3 d of exposure, nor in the fish from group TAM2 after 1 d of treatment. However, in group TAM2, degeneration of the epithelium of renal tubules was observed in all examined individuals after 10 d of exposure. Less numerous signs of epithelial degeneration were detected in three specimens from group TAM2 after 3 d of treatment, as well as in two fish from group TAM1 after 10 d of exposure (Fig. 4).

## Discussion

After 1 d of exposure to the tested herbicide, the experimental fish had significantly more misshapen erythrocytes. At the same time, no haematological changes in standardly measured red blood cell parameters (basic and derived) or white blood cell parameters were detected. This suggests that the shape of RBCs can be used as a rapidly responding marker of herbicide toxicity. Higher percentage of abnormally shaped erythrocytes was also detected in common carp treated with a clomazone-based herbicide formulation after 3 d of exposure (9). Fathy *et al*. (22) observed poikilocytosis in Nile tilapia (*Oreochromis niloticus*) exposed to herbicide formulations based on acetochlor, bispyribac-sodium, bentazon, halosulfuron-methyl, bensulfuron-methyl and quinclorac. Some other chemicals may also cause alterations in the shape of erythrocytes. For example, exposure of common carp to malachite green applied at a therapeutic concentration resulted in an increased percentage of RBCs exhibiting abnormal shape (7). Misshapen cells of this type were also observed in Prussian carp (*Carassius gibelio*) exposed to cadmium chloride (CdCl_2_ × 2.5H_2_O), zinc sulphate (ZnSO_4_ × 7H_2_O) and a mixture of these two substances (19). These results support the finding by Witeska (50) that fish erythrocytes are sensitive to environmental pollution and that the evaluation of their morphology can be used to assess chemical toxicity.

After 3 and 10 d of exposure, the fish showed significantly decreased MCH. These alterations were associated with small and often statistically insignificant changes in RBC count and HGB concentration, with their biological significance being difficult to determine. Our previous studies (9, 10) showed that fish of the same species exposed to a 2-methyl-4-chlorophenoxyacetic acid (MCPA)-based or clomazone-based herbicide formulation exhibited decreased MCH and MCV values, while MCHC was increased. Wintrobe’s indices (MCV, MCH and MCHC) were elevated in common carp treated with the glyphosate-based herbicide Roundup (8). Adene *et al*. (1) showed that African catfish (*Clarias gariepinus*) treated with nicosulfuron exhibited an increase in MCH and MCV, while MCHC was decreased. Other types of pesticides can also cause changes in derived erythrocyte indices. As demonstrated by Kole *et al*. (27), exposure of silver barb (*Barbonymus gonionotus*) to Sumithion 50EC (emulsifiable concentrate) – a formulation based on the insecticide fenitrothion – resulted in increased MCV and MCH values, while MCHC was more frequently elevated and less commonly decreased. A study conducted by Akter *et al*. (2) showed that climbing perch (*Anabas testudineus*) exposed to cypermethrin (10% EC) had decreased MCV and MCH, while MCHC either increased (more often) or decreased (less commonly). Atamanalp and Yanik (4) reported that rainbow trout (*Oncorhynchus mykiss*) exposed to the fungicide mancozeb showed a decrease in MCH value. These research findings suggest that changes in derived erythrocyte indices depend on both the type of herbicide applied and the fish species.

An increased RBC count and erythroblast percentage in the blood of experimental fish observed after 10 d of treatment indicate the release of immature erythrocytes from hematopoietic organs, likely accompanied by enhanced erythropoiesis. This may represent a compensatory response of the organism to increased oxygen demand related to intensified detoxification processes. However, the possibility that the increase in RBC count results from impaired gas exchange and tissue hypoxia cannot be excluded. Nevertheless, the absence of histopathological changes in the gills appears to contradict this supposition. It should be emphasised that the conclusion regarding increased erythropoiesis is preliminary and requires verification through the analysis of haematopoietic tissue activity. Haematopoietic analysis can provide insights into the mechanisms underlying the observed haematological changes and usefully complement standard haematological tests (52). Therefore, it seems justified to include this type of analysis in future experiments involving nicosulfuron-based herbicide formulations. Interestingly, Adene *et al*. (1) found an increase in RBC count in African catfish exposed to nicosulfuron. Thus, it can be assumed that an increase in erythrocyte count is a typical response in fish exposed to nicosulfuron and nicosulfuron-based herbicide formulations. To verify this assumption, however, further experiments involving different fish species are necessary. It should be noted that changes in RBC count may also result from exposure to other pesticides. For example, exposure of streaked prochilod (*Prochilodus lineatus*) to Roundup Transorb resulted in a higher count (36). On the other hand, cypermethrin (10% EC) caused a decrease in erythrocyte count in climbing perch (2). Carbendazim (50% WP (wettable powder)) treatment also led to a fall in RBC count in striped snakehead (*Channa striata*) (20). As can be seen, the results obtained by different authors vary. A fallen RBC count indicates an anaemic response, whereas a risen one suggests a compensatory response. According to Bojarski and Witeska (11), an anaemic response signifies adverse effects of pesticides on circulating erythrocytes and/or inhibition of erythropoiesis, while red blood cell parameters trending upwards may reflect a response to hypoxia.

All the differences in haematological parameters detected in the present study between the experimental groups and the control group were observed in fish exposed to Tamizan at the higher tested concentration. Exposure to the same agent at a lower concentration did not result in statistically significant changes. Furthermore, most of the statistically significant differences in haematological indices between the experimental group and the control group were noted after 10 d of exposure. Therefore, it can be concluded that the observed changes were both concentration- and time-dependent.

An increase in plasma glucose concentration in fish exposed to the tested herbicide at the higher concentration was observed after 1 d of treatment and persisted until the second sampling. Therefore, glucose concentration can be considered a reliable rapidly responding marker of Tamizan toxicity. A transient increase in glucose concentration was also observed in common carp exposed to Roundup after 1 d of treatment (8). Similarly, hyperglycaemia was observed in rohu (*Labeo rohita*) on the 2^nd^ day of exposure to the carbendazim-containing fungicide formulation Tagstin, whereas measurements taken on the 10^th^ and 15^th^ days revealed no significant differences between the experimental and control groups (28). Nevertheless, the elevation of blood glucose level in fish exposed to pesticides is not always a transient phenomenon. A study conducted by Sweilum (45) demonstrated that Nile tilapia subjected to the insecticide malathion exhibited significantly increased serum glucose concentration after 24 weeks of exposure. The rise in plasma glucose concentration noted in the present study in common carp exposed to Tamizan at the lower tested concentration occurred after 10 d of treatment. The observed stress response was therefore dependent on herbicide concentration and exposure duration. As shown in the review by Bojarski *et al*. (12), increases in blood glucose concentration in fish exposed to various toxic agents were observed approximately three times more frequently than decreases. This observation suggests that elevated glucose level is a typical symptom of chemical stress in fish (12).

The reduction in total cholesterol concentration in fish blood plasma observed in the current study was noted only after 1 d of exposure. Such temporary changes may suggest a nonspecific response of which the mechanism remains uncertain. It cannot be ruled out that the lower cholesterol concentration was related to the inhibition of the expression of genes responsible for cholesterol synthesis, since it has been demonstrated that some pesticides show such effects. For example, Uren Webster *et al*. (47) demonstrated that the herbicide linuron caused inhibition of cholesterol biosynthesis in brown trout (*Salmo trutta*) and hypothesised that it might result from the disruption of androgen signalling. Mu *et al*. (37) showed that the fungicide difenoconazole inhibited cholesterol-genesis gene expression and reduced hepatic cholesterol level in the liver of male zebrafish.

In the current study, histopathological changes were observed only in the trunk kidneys of exposed fish, while the gills and livers remained unaffected. The renal epithelium of trunk kidneys of fish intoxicated with Tamizan at the higher tested concentration for 10 d showed degeneration. After 3 d of exposure, such changes were also visible, although to a much lesser extent. These findings suggest that exposure to the tested herbicide formulation at a concentration corresponding to 5 mg/L of the active ingredient (nicosulfuron) for longer than a few days has a pronounced negative effect on the trunk kidney of common carp. Moreover, signs of degeneration of the epithelium of renal tubules were also found in some fish exposed to the herbicide at the lower tested concentration, but only after 10 d of treatment. Therefore, it can be concluded that the observed histopathological changes were clearly both time- and concentration-dependent. Interestingly, the histopathological changes in the trunk kidney of fish detected in the present study were confined to the renal tubules, while the renal corpuscles appeared unaffected.

Degeneration of renal tubule epithelium is one of several common pathological changes and was frequently observed in fish exposed to various substances such as lead acetate (38), the antimalarial drug chloroquine (41), a chlorpyrifos-based insecticide formulation (34) and a mancozeb-based fungicide formulation (15). The present study did not reveal any other types of lesions. In our previous studies, the effect of herbicide formulations not based on nicosulfuron on common carp organs was tested using methods similar to those applied in the current research. When the toxicant was Roundup, no histopathological changes were detected; however, a group exposed to a high Roundup concentration for 10 d was not examined histologically because group mortality was 100% (8). Similarly, fish exposed to Chwastox, an MCPA-based herbicide formulation, did not exhibit histopathological changes in the gills, liver or trunk kidney (10). In these two studies, concentrations equivalent to 1 and 5 mg/L of the active substance were used, as in the present experiment. Therefore, the present results may suggest that nicosulfuron is more nephrotoxic than glyphosate or MCPA. However, additional studies employing more sophisticated histological techniques are required to confirm this conclusion.

The limitations of the present study include the experiment’s single fish species, omission of aquatic invertebrates, the use of no advanced histological techniques or molecular biology tools, and the short duration of herbicide exposure.

## Conclusion

This study demonstrates that exposure of common carp to Tamizan, a nicosulfuron-based herbicide, may induce functional (pathophysiological) as well as structural (histopathological) changes. The observed changes suggest that the percentage of misshapen erythrocytes and the plasma glucose concentration can be considered rapidly responding biomarkers of the toxic effect induced by Tamizan. The recorded changes in haematological and blood biochemical parameters may indicate possible erythrocyte damage, a compensatory response (release of erythrocytes into the bloodstream and likely enhanced erythropoiesis), a stress reaction and possible disturbances in cholesterol/lipid metabolism. Alterations in the microstructure of the trunk kidney (degeneration of the renal tubules) suggest nephrotoxicity of the tested herbicide formulation. The changes noted in the present study were generally time- and concentration-dependent. Understanding the precise mechanisms underlying these changes requires further research. Since nicosulfuron can be toxic to aquatic organisms, the monitoring of aquatic ecosystems for the presence of this agent should be carried out.
